# Hepatitis E virus genotypes and subgenotypes causing acute hepatitis, Bulgaria, 2013–2015

**DOI:** 10.1371/journal.pone.0198045

**Published:** 2018-06-07

**Authors:** Roberto Bruni, Umbertina Villano, Michele Equestre, Paola Chionne, Elisabetta Madonna, Dilyana Trandeva-Bankova, Maria Peleva-Pishmisheva, Tencho Tenev, Eleonora Cella, Massimo Ciccozzi, Giulio Pisani, Elitsa Golkocheva-Markova, Anna Rita Ciccaglione

**Affiliations:** 1 Viral Hepatitis and Oncovirus and Retrovirus Diseases Unit, Department of Infectious Diseases, Istituto Superiore di Sanità, Rome, Italy; 2 Department of Neurosciences, Istituto Superiore di Sanità, Rome, Italy; 3 Department of Virology, National Center of Infectious and Parasitic Diseases, Sofia, Bulgaria; 4 Department of Infectious Diseases, Regional Hospital-Pazardzhik, Pazardzhik, Bulgaria; 5 Public Health and Infectious Diseases, Sapienza University, Rome, Italy; 6 Unit of Clinical Laboratory Science, Campus Bio-Medico University, Rome, Italy; 7 Biologicals and Biotechnologicals Unit, National Centre for the Control and Evaluation of Medicines, Istituto Superiore di Sanità, Rome, Italy; Centers for Disease Control and Prevention, UNITED STATES

## Abstract

**Background:**

In industrialized areas of the world, including Europe, Hepatitis E Virus (HEV) is considered an emerging pathogen. In fact, autochthonous cases caused by HEV genotype 3 (HEV-3) are increasingly reported. Several studies described the human HEV-3 subtypes and strains circulating in West Europe countries; in contrast, very little is known about the HEV strains responsible for acute hepatitis E in countries of East Europe/Balkans, such as Bulgaria.

**Methods and findings:**

Anti-HEV IgM positive serum samples (n = 103) from acute hepatitis cases (2013–2015) from all over Bulgaria were analysed for HEV RNA by Real-Time PCR. Viremia was detected in 90/103 samples. A fragment of the viral genome (ORF-2 region) was amplified by nested PCR from 76/90 viremic samples, leading to a sequence in 64 of them. Genotyping by phylogenetic analysis with standard reference sequences showed HEV-1 in 1/64 cases, HEV-3 in 63/64. Subtyping of HEV-3 sequences showed 3e (39/63, 62%), 3f (n = 15/63, 24%) and 3c (n = 8/63, 13%) subtypes; in one case the sequence subtype was uncertain and classified as 3hi. In the phylogenetic tree, most 3e sequences grouped in two well distinct clusters (A and B), each one with very low intragroup genetic distances. In contrast, 3f and 3c were interspersed with reference sequences and showed lower tendency to cluster and/or higher intragroup distances. Geographically, while 3f and 3c were scattered throughout the country, 3e was restricted to the South-West area, with most cases in two towns about 40 kilometres apart from each other.

**Conclusions:**

Most acute hepatitis E cases in Bulgaria are caused by HEV-3, subtypes 3e, 3f and 3c. Circulation of 3e appears quite different from 3f and 3c, with 3e restricted to the South-West area while 3f and 3c diffused over the country. The factors underlying the observed molecular and geographical differences remain to be investigated.

## Background

The Hepatitis E Virus (HEV) belongs to the *Hepeviridae* family. According to a recent consensus for classification, HEVs found in humans are sub-classified in the genus *Orthohepevirus*, species *Orthohepevirus A*, which also includes viruses infecting animal species [1]. Molecular characterization of the HEV genome from several human and animal samples has shown *Orthohepevirus A* viruses can be grouped into 8 genotypes (HEV-1 to HEV-8), further subdivided in subtypes. The latest genotype (HEV-8) was discovered in Bactrian camels from Xinjiang, China [2]. To date, it is well established that genotype 1, 2, 3 and 4 may infect humans; a single case of infection by genotype 7 has also been reported, but it is unknown if this genotype is regularly transmitted to humans or its transmission may occur only in exceptional circumstances [3].

The epidemiology of HEV-1 and HEV-2 appears to be quite different from that of HEV-3 and HEV-4 [4]. In fact, HEV-1 and HEV-2 infect exclusively humans and circulate in Asia and Africa, show fecal-oral transmission (mostly through contaminated water) and are frequently responsible for HEV outbreaks in those areas; in western countries, these genotypes are responsible for sporadic cases related to travel in countries in which HEV-1 and HEV-2 are endemic. In contrast, HEV-3 and HEV-4 circulate in both humans and animal species and are believed in most cases to be transmitted to humans as a zoonosis. While HEV-4 has been found in sporadic cases of acute hepatitis E from East Asia [5], HEV-3 occurs worldwide. It is estimated that most HEV-3 transmissions cause no symptoms and only in a fraction of cases infection is responsible for clinically evident acute hepatitis; this is also shown by data from an outbreak on a cruise ship [6].

In Europe, HEV-3 is considered an emerging pathogen and there is much debate about the circulation of autochthonous HEV-3 strains, as shown by the results from several studies in blood donors and in acute hepatitis cases [7–10].

To obtain a comprehensive overview of the circulation at European level, a better knowledge of the HEV epidemiology as well as of the subtypes and strains responsible for acute hepatitis E in each European country is needed.

Limited information is available about HEV epidemiology in Bulgaria, and even less about the circulating HEV genotype/subtypes and strains. A small study conducted in 2010–2012 revealed that 44% (14/32) of patients with non-A non-C acute hepatitis were positive for HEV. Sequence analysis showed infection by HEV genotype 3, demonstrating that autochthonous HEV infection occurs in Bulgaria too, as found in other European countries [11]. Two further studies estimated the fraction of HEV infections in surveys of acute hepatitis cases: in the first study, 2.48% of 806 hospitalized acute hepatitis cases were identified as acute HEV infections [12]; in the other study, 13.2% of 325 patients with acute hepatitis from North Eastern Bulgaria proved to be anti-HEV IgM positive [13]. Finally, a further study analysed the anti-HEV IgG prevalence in a convenience population (741 individuals undergoing ambulatory examination, due to various reasons), whose samples were collected in 2012 and 2013 in the Plovdiv region of the country. An overall anti-HEV IgG mean prevalence of 9.04% was found; no significant differences were observed between males and females and the prevalence increased with increasing age [14].

In the present study, the circulation of HEV genotypes, subtypes and strains in Bulgaria was investigated by analysing serum samples from acute hepatitis E cases occurred between January 2013 and May 2015 throughout the country. The data add a further piece to the puzzle of HEV subtypes and strains circulating in Europe.

## Materials and methods

### Patients

The Department of Virology of the National Center of Infectious and Parasitic Diseases in Sofia, Bulgaria, receives serum samples from patients with acute hepatitis from all over the country for laboratory testing. Patients are classified as acute hepatitis according to the clinical definition by World Health Organization (WHO) [15].

One hundred and three sera collected at the time of hospital admission from January 2013 to May 2015 in several towns/villages from acute hepatitis cases and found to be anti-HEV IgM positive were available for the present study. Anti-HEV IgM antibodies had been detected by the DIA.PRO HEV IgM assay. All cases were classified as confirmed HEV infections on the basis of specific IgM antibody detection, according to the case definition by WHO. Among them, 87/103 (84%) were males, the mean age was 57 years, the median age was 58 years (range: 14–84 years). A major fraction of cases was from three cities/towns: Sofia (23 cases, mean age: 58 years, males: 87%); Plovdiv (32 cases, mean age: 56 years, males: 81%); Pazardzhik (28 cases, mean age: 59 years, males: 82%). For most remaining towns only 1 sample was available, with a few exceptions (Ruse, Samokov, Pleven: 2 samples/town; Dobritch: 3 samples/town).

Serum HEV RNA quantification and characterization by nested RT-PCR and sequencing was carried out in Italy at the National Reference Laboratory for Hepatitis Viruses—Istituto Superiore di Sanità (NRL-ISS) (see next paragraph). Written informed consent for participation in the study had been obtained before patient discharge. The study was approved by the Ethics Committee at the National Center of Infectious and Parasitic Diseases, Sofia, Bulgaria.

### Serum HEV RNA quantification by Real-Time PCR

Nucleic acids were extracted from 200 μL serum by the Qiamp MinElute Virus Spin kit (QIAGEN). HEV RNA quantification was carried out by an *in house* Real-Time PCR method, using previously reported primers [16]. A 70 bp region of the ORF3 (position 5261–5330 on full length HEV genome sequence Acc.N. M73218) was amplified from 7 μL RNA extract (equivalent to RNA extracted from 35 μL serum). Amplification was carried out on the Rotor-Gene Q 5plex Platform (QIAGEN, Hilden, Germany) by the QuantiTect Probe RT-PCR kit (QIAGEN, Hilden, Germany). Data were analysed by the Rotor-Gene Q software, Version 2.1.0 (Build 9). The analytical sensitivity of the validated method is 35–40 IU/mL HEV RNA (95% detection limit).

### Nested RT-PCR and sequencing

Viral RNA was extracted from 200 μL serum by using the QIAmp MinElute Virus Spin kit (Qiagen, Hilden, Germany). One seventh extracted RNA (5 μL) was reverse transcribed by the SuperScript III First-Strand Synthesis System for RT-PCR (Invitrogen) with random hexamers. Nested PCR was carried out with primer sequences made available by RIVM to European laboratories participating to the HEVnet database. For the first PCR step, the forward primer was from positions 5909–5934 of the HEV genome sequence (Accession Number M73218) and the reverse primer was complementary to positions 6512–6534; PCR cycling conditions were as follows: 35 cycles of 95°C 30sec; 42°C 30 sec; 60°C 45 sec. For the second (nested) PCR, the forward primer was from positions 5948–5985, while the reverse primer was complementary to positions 6479–6513; PCR cycling conditions were as follows: initial denaturation 95° 6 min, then 40 cycles of 95°C 30sec; 60°C 20 sec; 72° C 15 sec. The presence and size of amplification products were assessed by agarose gel electrophoresis.

To control for PCR contamination, negative control sera were extracted and subjected to reverse transcription and nested PCR in each run, along with the sera to be assayed. Sequencing of purified PCR products was carried out by using the GenomeLab Dye Terminator Cycle Sequencing (DTCS) Quick Start Kit and an automated DNA sequencer (both kit and instrument by Beckman Coulter, Inc., Fullerton, CA). The sequencing reaction was carried out according to the manufacturer instructions. The output sequences were aligned by ClustalW in BioEdit [17].

A region shared by the aligned sequences was finally selected for phylogenetic analysis: it encompassed a 355 nt fragment of the ORF2 region of HEV genome (positions 6120 to 6474 of the reference sequence Acc.No. M73218). The sequences of the present study were deposited in GenBank (Accession number MH203164 to MH203227).

### Sequence analysis

HEV genotyping and subtyping were carried out as previously proposed in a previous publication, to allow results to be compared with other studies [16]. In particular, the analysis included the recommended standard reference sequences and the same phylogenetic approach used in previous studies [18,19].

HEV genotype was assigned by phylogenetic analysis of a dataset containing the Bulgarian HEV sequences under study (n = 64) *plus* the recommended set of standard reference HEV sequences [18]. Then, the identified HEV-3 sequences (n = 63) were sub-typed by phylogenetic analysis of a dataset including the recommended HEV-3 subtype references (3a, 3b, 3c, 3e, 3f, 3g, 3h, 3i, 3j) as well as some additional previously subtyped strains [18,19].

Phylogenetic relationships were analysed by constructing a Neighbor-Joining phylogenetic tree calculated on the Maximum Composite Likelihood distances by MEGA 6.0 [20], as in previous studies [18,19]. Tree reliability was assessed by setting bootstrap replicates to 1000. Bootstrap values > 70 were considered significant.

Mean intragroup corrected distances were estimated by the Maximum Composite Likelihood method in Mega 6.0, as previously reported [19].

### Statistical analysis

Simple statistical analysis was used to estimate if observed variations of serum virus concentration (quantified as HEV RNA IU/mL) were correlated with age (Pearson correlation) or associated with gender, HEV subtype, sequence group (T-test).

## Results

### HEV RNA quantification and identification of HEV RNA positive samples

Real-Time PCR showed variable levels of viremia in 90/103 (87%) anti-HEV IgM positive samples. Median viral load was 1.2x10^5^ IU/mL, range: 1.3x10^3^-8.5x10^6^ IU/mL. The magnitude of viral load showed no significant differences between males and females and no significant correlation with age or the different HEV-3 subtypes (see below for description of identified HEV-3 subtypes). The fraction of HEV RNA positive samples at the town level (measurable only for three towns with several samples, see Materials and methods) did not show significant differences: Sofia 19/23 (82.6%), Plovdiv 28/32 (87.5%), Pazardzhik 25/28 (89.3%).

### Genotyping and subtyping

To obtain a PCR product for sequencing, Real-Time PCR positive samples were subjected to nested RT-PCR, that was successful in 76/90 (84%) of them. A sequence was finally obtained from 64 samples.

**Fig 1:**
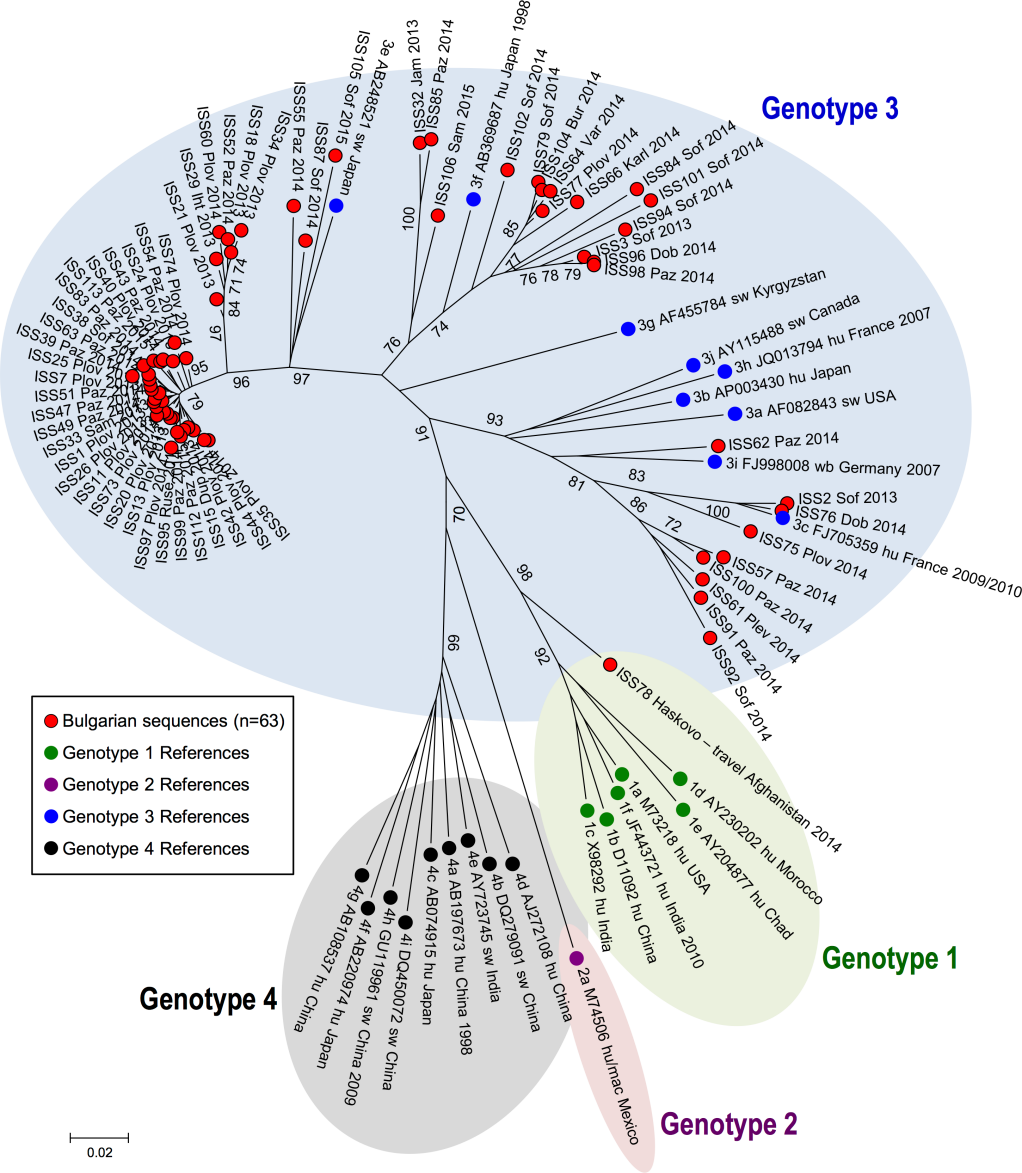
Phylogenetic tree of the 64 Bulgarian HEV sequences. A red circle marks Bulgarian sequences; for each sequence, the town/village (Dup: Dupnica; Jam: Jambol; Iht: Ihtiman; Karl: Karlovo; Paz: Pazardzhik; Plev: Pleven; Plov: Plovdiv; Sam: Samokov; Sof: Sofia; Var: Varna) and the isolation year (2013, 2014, 2015) are reported after the sequence ID. Reference strains with known genotype (1, 2, 3 and 4) were included in the analysis and are marked as shown in the box. For each reference sequence, genotype/subtype (e.g. 3e), Accession number, host (hu: human; sw: swine), country and year of detection are reported. Significant bootstrap values (>70) are reported.

Fig 1 shows the results of phylogenetic analysis of the 64 HEV sequences from Bulgaria and 25 reference sequences. The phylogenetic tree revealed that 63 of them were genotype 3; one isolate was genotype 1. This latter sequence was obtained from a refugee from Afghanistan who was accommodated in a transit centre, located near the Bulgarian-Turkish border. No genotype 2 or genotype 4 isolates were observed.

**Fig 2:**
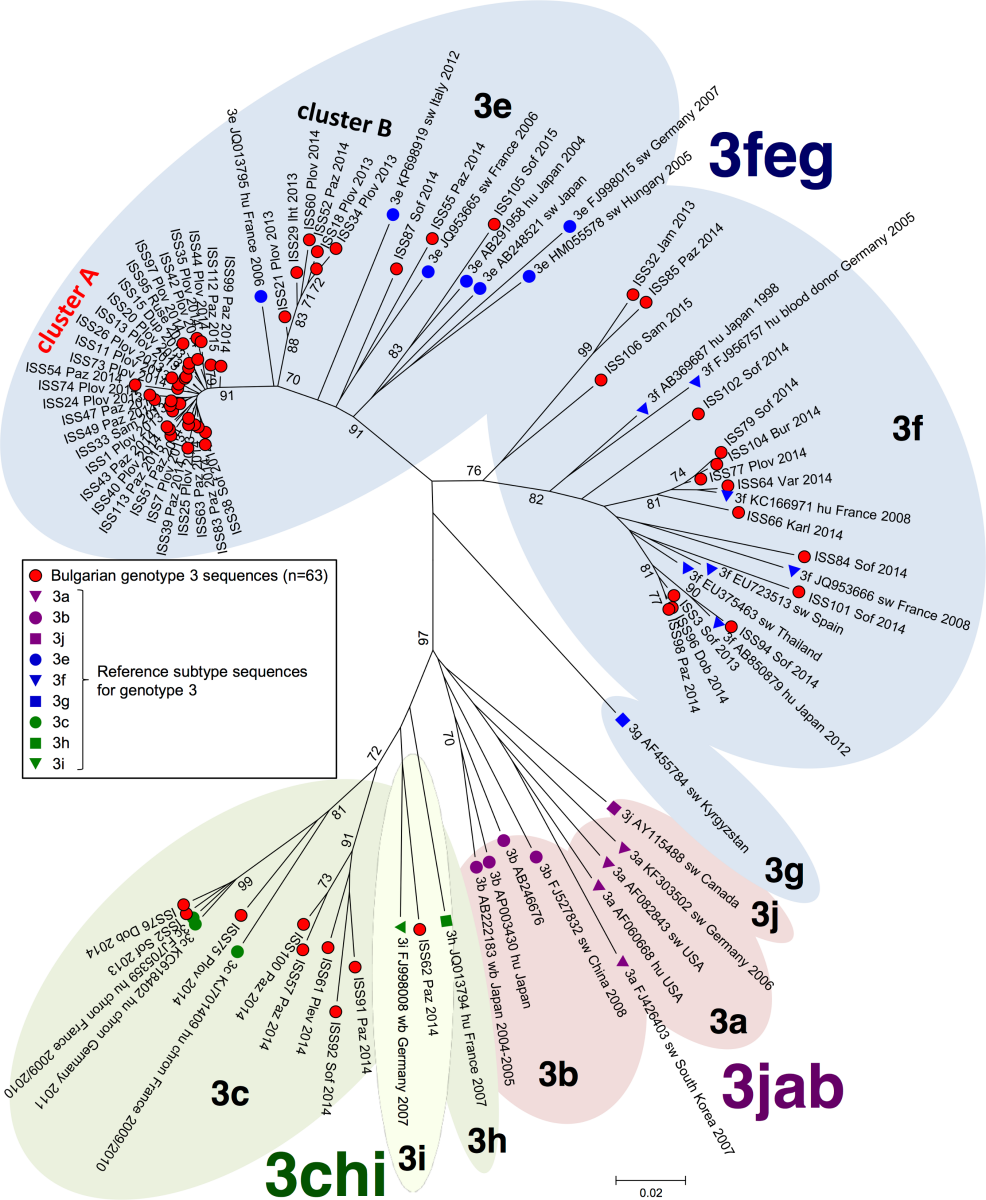
Phylogenetic tree of the 63 HEV genotype 3 isolates from Bulgaria. A red circle marks Bulgarian sequences; for each sequence, the town/village (Dup: Dupnica; Jam: Jambol; Iht: Ihtiman; Karl: Karlovo; Paz: Pazardzhik; Plev: Pleven; Plov: Plovdiv; Sam: Samokov; Sof: Sofia; Var: Varna) and the isolation year (2013, 2014, 2015) are reported after the sequence ID. Reference genotype 3 strains with known subtype were included in the analysis and are marked as shown in the box. For each reference sequence, genotype and subtype (e.g. 3e), Accession number, host (hu: human; sw: swine), country and year of detection are reported. Significant bootstrap values (>70) are reported.

Fig 2 reports the subtyping results by phylogenetic analysis of the 63 HEV-3 Bulgarian isolates along with subtype 3 references. The tree shows an overall structure with three major groups (3feg, 3jab, 3chi), in agreement with previous reports analysing both complete genome sequences and partial ORF2 sequences smaller (187 nt) than those of the present study (355 nt) [19].

The majority of Bulgarian HEV-3 sequences grouped in the statistically well supported 3e, 3f and 3c branches; thus, they could be safely assigned to subtype 3e (39/63, 62%), 3f (n = 15/63, 24%) and 3c (n = 8/63, 13%). Unique exception was the sequence ISS62 Paz 2014: it was located in the 3chi clade and clustered with the reference 3i sequence, but the bootstrap values at the nodes separating the 3i and 3h references were not significant. Thus, the ISS62 Paz 2014 isolate was finally classified as subtype 3hi, to indicate uncertain subtype assignment.

A surprising result was that most Bulgarian subtype 3e sequences (36/39, 92%) grouped in two sub-clusters in the 3e sub-tree: a main subcluster (n = 30 sequences) and a minor one (n = 6 sequences), labelled "Cluster A" and "Cluster B" in Fig 2, respectively. In each cluster, the sequences are highly related. Significantly, most cases from these two clusters were from a narrow geographical area (see next paragraph).

### Geographical distribution of the detected subtypes and strains

**Fig 3:**
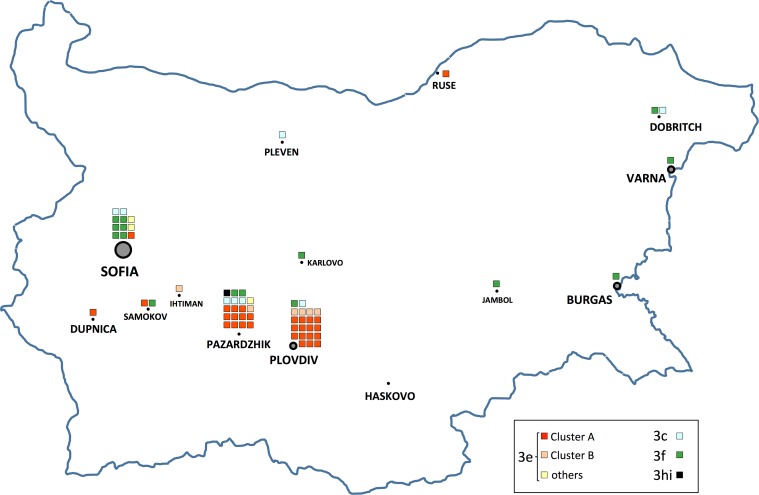
Geographical localization of the characterized HEV genotype 3 strains in Bulgarian towns/villages. Cases are represented by a colour coded square, according to the box included in the figure. The Haskovo town, in which a patient infected by HEV genotype 1 was identified, is also reported.

Fig 3 reports the geographical distribution of the detected subtypes and strains in Bulgaria, according to the town of residence of the patients. All but one 3e sequences were detected in the South-West region of the country, mostly in two towns: Plovdiv and Pazardzhik. More in detail, 26/30 (87%) 3e sequences from cluster A were detected either in Plovdiv (n = 15) or in Pazardzhik (n = 11), and a similar distribution was also shown by cases from the small cluster B, with 5/6 cases having occurred either in Plovdiv (n = 4) or in Pazardzhik (n = 1). Among the five other towns in which 3e sequences from cluster A and B were detected, four of them were located West of Pazardzhik and Plovdiv (Sofia, Dupnica and Samokov for cluster A, Ihtiman for cluster B); the fifth case was observed far away, in Ruse, in the Northern part of Bulgaria. In striking contrast with most 3e cases, the 3c and 3f subtypes were scattered throughout different towns of the country.

### Distance analysis

Analysis of the genetic distances of Bulgarian 3e sequences showed that the mean intragroup corrected distances of sequences in Cluster A (n = 30) and B (n = 6) were 0.015 and 0.019, respectively. In contrast, it was 0.114 in the other 3e Bulgarian sequences (n = 3) and 0.111 in the 3e references included in the present study (n = 7); these latter values are in agreement with previously published intra-subtype corrected distances of HEV-3 subtypes (range: 0.090 to 0.145) [19]. Thus, the mean intragroup distances in cluster A and cluster B were about 6–7 fold lower than the typical intra-subtype values found in genotype 3.

Analysis of the two other subtypes observed in the present study showed that the mean intragroup distances of Bulgarian 3c (n = 8) and 3f (n = 15) sequences, collected from all over the country, were 0.089 and 0.085, respectively; to check for possible local differences, the analysis was also restricted to the 3c and 3f sequences detected in the same South West area of 3e cluster A and B (Fig 3: 3c from Sofia, Pazardzhik, Plovdiv, n = 6; 3f from Sofia, Pazardzhik, Plovdiv, Samokov and Karlovo, n = 11), but the distance values were 0.085 and 0.091, respectively, i.e. substantially similar.

### Temporal distribution of cases with cluster A and B sequences

**Fig 4:**
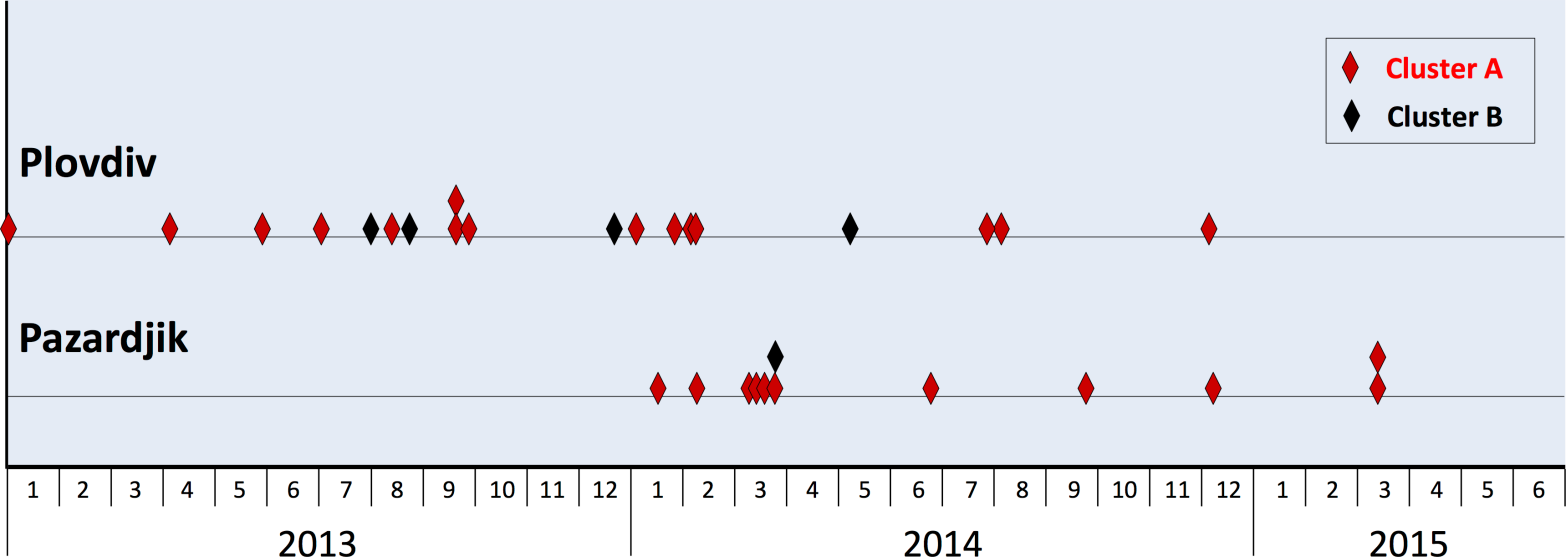
Onset date of cases, from Plovdiv and Pazardzhik, harbouring cluster A and cluster B sequences, 3e subtype. Cases from cluster A and B (see Fig 2) are indicated according to the box included in the figure.

Fig 4 reports graphically the sample collection dates of 3e sequences in cluster A and cluster B from Plovdiv (n = 15 and 4, respectively) and Pazardzhik (n = 11 and 1, respectively). In 2013 these sequences were detected exclusively in Plovdiv; in Pazardzhik, they were first detected an year later, on January 2014 (cluster A) and March 2014 (cluster B). The first detection of either cluster A or B sequences in any other towns (not reported in Fig 4) was on July 2013 (Dupnica) or later (Ihtiman; October 2013; Samokov: December 2013; Sofia: January 2014; Ruse: November 2014).

## Discussion

The present study reports the description of HEV subtypes and strains found in a survey of anti-HEV IgM positive acute hepatitis E cases occurred in Bulgaria in 2013, 2014 and early 2015.

Most samples showed detectable HEV RNA by Real-Time PCR. The wide range of observed virus concentration (about 4 log_10_) was possibly due to the expected variable time between onset of symptoms and sampling of serum as well as to individual virus/host variability. In fact, virus concentration was not statistically associated or correlated to any tested variables (gender, age, subtype).

In 14 HEV RNA positive samples (16%), the attempt to obtain a PCR product suitable for sequencing was not successful. For seven of them, the lack of amplification could be explained as a result of the lower viremia (< 7,000 IU/mL) combined with the lower sensitivity of nested-PCR than Real-Time PCR; other unknown reasons could be involved in the remaining samples, such as variability in the ORF2 sequence fragments targeted by nested PCR primers, resulting in poor annealing and no amplification (less likely in the more conserved ORF3 regions targeted by Real-Time PCR primers), or presence of RT-PCR inhibitors in the samples or a combination of them.

The sequencing data of 64 cases show that acute hepatitis E cases in Bulgaria are usually due to HEV genotype 3: only 1/64 cases harboured HEV genotype 1. This finding is in agreement with this patient being a refugee from Afghanistan, who was accommodated in a transit center: HEV-1 is endemic in Middle East countries [7], thus infection was likely acquired in his country of origin some weeks before the onset of symptoms.

At the subtype level, the 3e, 3f, 3c and 3hi were detected in the present survey, although at different frequencies, i.e. the same subtypes observed in West Europe countries [21,22]. The 3e subtype was the most frequent (61%) and 3e *plus* 3f accounted cumulatively for the majority of genotype 3 cases (85%).

The sequences of most 3e cases grouped in two phylogenetically well distinct clusters, each of them with very low mean intragroup distance. These lineages were well separated even from the most closely related sequence detected in GenBank by BLAST search (Fig 2: sequence Acc.No. JQ013795). The members of both clusters shared narrow geographical distribution: except one case in the North of the country, they were from South-West Bulgaria, mainly from two towns (Plovdiv and Pazardzhik) about 40 kilometres apart from each other (Fig 3). Plovdiv is one of the biggest cities in Bulgaria and is about 5-fold bigger than the nearby Pazardzhik: it is known that many people living in Pazardzhik move daily to Plovdiv to work and, thus, may have the opportunity to share unknown infection sources, potentially explaining why most cluster A and B cases were from both these centers. The single case observed in Ruse, in the North of the country, might have acquired infection by travel in the South West region; unfortunately, no data were collected that could support this hypothesis. Alternatively, this case raises the possibility that cluster A strains may be diffused in other regions of the country too, but are actually missed because of misdiagnosis or poor sampling from these regions. Further future studies will have to consider this latter possibility and promote active search for cases in rural regions to improve the reliability of observed country wide circulation of HEV strains.

In terms of time, while in Plovdiv the strains of cluster A and B were detected since early 2013, in Pazardzhik the first cases appeared an year later, in 2014; the first detection in any other towns was on July 2013 (1 case in Dupnica) (Fig 4). These findings suggest the hypothesis that circulation of cluster A and B strains in the South West area may have started in Plovdiv and then, in mid- and late-2013, spread to other towns of the area, mainly to Pazardzhik; unfortunately, this interpretation is limited by the moderate sample size and by the lack of samples collected earlier than 2013. Even with these limitations, the low variability, the narrow geographical distribution and the onset dates are consistent with highly localised circulation of HEV genotype 3e strains in the Plovdiv and Pazardzhik area, resembling more a localised outbreak rather than sporadic transmission cases.

In striking contrast, 3c and 3f sequences of the present survey showed both higher intra-subtype variability and wider distribution over the country than 3e. The factors underlying the observed differences between 3e *vs*. 3c and 3f remain, at present, unknown and further investigations are needed to identify them. Clearly, surveillance improvement and larger sampling may contribute to obtain a more accurate description of the HEV subtype circulation in Bulgaria.

A limitation of the present study is the lack of data about HEV strains circulating in swines in Bulgaria: in fact, consumption of undercooked pork is the most commonly reported route of HEV-3 infection. Analysis of HEV-3 circulating in swine samples from pig farms supplying Plovdiv and/or Pazardzhik could confirm or deny the swine source of the human clusters.

## Conclusions

This is the first study reporting an overview of HEV subtypes and strains circulating in acute hepatitis E cases in Bulgaria, helpful to delineate a more comprehensive picture of the HEV circulation in Europe.

HEV genotype 3 was responsible for all but one detected hepatitis E cases in 2013, 2014 and early 2015 and 3e, 3f and 3c were the most frequent subtypes. Further future studies should be carried out to monitor any variation of the relative frequency and circulation of these subtypes in Bulgaria, possibly linked to variation in transmission pathways. The molecular characterisation will prove especially useful to investigate the factors underlying the spread of HEV infections, particularly in the South-West area of the country where most cases occurred, to implement appropriate preventing measures.
